# Combined neutrophil/platelet/lymphocyte/differentiation score predicts chemosensitivity in advanced gastric cancer

**DOI:** 10.1186/s12885-018-4414-6

**Published:** 2018-05-02

**Authors:** Zhenhua Huang, Yantan Liu, Chen Yang, Xiaoyin Li, Changqie Pan, Jinjun Rao, Nailin Li, Wangjun Liao, Li Lin

**Affiliations:** 1 Department of Oncology, Nanfang Hospital, Southern Medical University, Guangzhou, 510515 China; 2 Key laboratory of new drug screening of Guangdong Province, School of Pharmaceutical Sciences, Southern Medical University, Guangzhou, China; 3 Department of Medicine-Solna, Karolinska Institute, Clinical Pharmacology Group, Karolinska University Hospital-Solna, Stockholm, Sweden

**Keywords:** Gastric cancer, Chemosensitivity, Neutrophil-to-lymphocyte ratio, Platelet-to-lymphocyte ratio, Tumor differentiation

## Abstract

**Background:**

Gastric cancer is common in developing regions, and we hope to find out an economical but practical prognostic indicator. It was reported that pre-treatment peripheral neutrophil-to-lymphocyte ratio (NLR) and platelet-to-lymphocyte ratio (PLR), as well as differentiation status, were associated with cancer progression. Hence, we introduced a novel combined Neutrophil/platelet/lymphocyte/differentiation Score (cNPLDS) to improve the prediction value of palliative chemotherapeutic response in advanced gastric cancer.

**Methods:**

According to statistical sample size estimation, 136 primary diagnosed unresectable advanced ptaients were included for a retrospective study. The follow-up end-point was progression free survival (PFS) during the first-line palliative chemotherapy. Differentiation stratified patients into well, medium and poor groups by score 1 to 3, while patients with neither elevated NLR and PLR, only one elevated, or both elevated were of the combined NLR-PLR score (cNPS) 1 to 3, respectively. The cNPLDS was calculated by multiplying the tumor differentiation score and cNPS.

**Results:**

Determined by the receiver operating characteristic (ROC) curve, the optimal cut-off points for NLR and PLR were 3.04 and 223. Through univariate analysis and survival analysis, poor differentiation, high NLR, high PLR, high cNPS, and high cNPLDS respectively indicated inferior PFS during the first-line palliative chemotherapy. Patients were furhter classified into low to high risk groups by cNPLDS. Groups of elevated NLR, PLR, cNPS, and cNPLDS showed lower disease control rate. Compared to other parameters, cNPLDS significantly improved the accuracy in predicing the first-progression.

**Conclusions:**

This study indicates that the novel parameter cNPLDS is superior to NLR or PLR alone, or even cNPS, in predicting the first-line chemosensitivity in advanced gastric cancer.

## Background

Gastric cancer is one of the most common malignant tumors in east Asia. In China, the 5-year survival rate of this disease is relatively lower because a large proportion of gastric cancer patients were already at advanced stage when diagnosis [1], and about 500,000 people died from it during the year 2015 [2]. At present, the major treatment option for advanced unresectable gastric cancer is chemotherapy. Unfortunately, doctors have to face that many patients give up further treatments just because of the economic reasons in the less developed regions. Previous studies have demonstrated that some cancer endogenous factors, such as differentiation status [3, 4] or certain genes expression [4–6], may influence the efficiency of chemotherapy. However, we still know very little about whether there are other affordable pre-treatment factors can better predict progression free survival in advanced unresectable gastric cancer patients, which may help to optimize the treatment strategies.

It was reported that systemic inflammatory response plays important roles in the progression of various cancers [7–9]. Circulating inflammatory cells can release many biological active factors and thus lead to tumor growth, progression, and metastasis [10–12]. Previously, we have demonstrated a close cooperation between neutrophils, platelets and lymphocytes in basic experiments [13, 14]. In clinical studies, neutrophil-to-lymphocyte ratio (NLR) and platelet-to-lymphocyte ratio (PLR) were adopted for prognosis evaluation in many cancers [15–23]. Recently, a study showed that preoperative combined NLR and PLR Score (cNPS) better predicted the postoperative survival in early stage gastric cancer than NLR or PLR alone [21]. However, it remains unclear whether cNPS has better prognostic prediction value for the first-line chemotherapy in advanced unresectable gastric cancer patients. Moreover, cNPS only implied the systemic inflammatory status. We suppose that taking the endogenous factor (e.g. differentiation status) into account together may further improve the prediction efficiency.

Therefore, we introduce a novel combined Neutrophil/platelet/lymphocyte/differentiation Score (cNPLDS), together with cNPS and other clinical indices, to find out the optimum parameters to predict survival and clinical responses of first-line chemotherapy for advanced gastric cancer.

## Methods

### Sample size estimation

Sample was estimated by statistical methods (http://powerandsamplesize.com/). Using the Cox PH, 2-Sided Equality Model online, the inputted parameters of estimated Hazard Ratio was 1.75 to 2.25 according to pervious similar study on advanced GC [16], the overall probability of event was 0.8, proportion of samples in groups was 1:1. As a result, the estimated sample size ranged from 60 to 126 by the power of 0.8.

### Patients and eligibility criteria

A retrospective analysis was performed based on the medical records at Nanfang Hospital, Guangzhou, China. From February, 2011 to August, 2017 at this center, there were 589 advanced gastric cancer patients who lost the radical resection opportunity. Among them, we only focused on the patients who initially treated with standardized palliative first-line chemotherapy with regular follow-up. Those patients who initially received second or more advanced line chemotherapy at our hospital were excluded from the study.

All the patients should have a Zubrod-ECOG (WHO) score ≤ 2. The histopathology diagnosis of gastric cancer was confirmed by at least two experienced pathologists in our hospital. According to WHO classification of stomach cancer histologic types, well differentiated is defined as being composed of well-differentiated adenoid structures, and intestinal metaplasia sometimes could be seen. Poorly differentiated is defined as being composed of irregular-differentiated glands and sometimes even hard to identify the adenoid structures. The microscopy appearance of moderately differentiated is between those of well differentiated and poorly differentiated. Patients had no history of hematological diseases. Antineoplastic therapies (chemotherapy, radiotherapy or immunotherapy, etc.) were not received within two months before the start of first-line chemotherapy, and other treatments that could affect bone marrow hematopoiesis were also not received before the start of first-line chemotherapy. The chemotherapy regimens were limited to the protocol of advanced gastric cancer recommended by guidelines of the Chinese Society of Clinical Oncology (CSCO), the National Comprehensive Cancer Network (US) (NCCN) and the European Society of Medical Oncology (ESMO). The patients changing the chemotherapy regimen before meeting the PD standard, or simultaneously receiving other antineoplastic therapy (including radiotherapy or immunotherapy, etc.) during the treatment, were excluded from the study. As a result, a total of 136 patients met the eligibility criteria and were included in the studies.

### Follow-up and assessment

The response to treatment was assessed about every two cycles of three-week regimens or every four cycles of two-week regimens (i.e., every 6 to 8 weeks) on the basis of the rules established by the Response Evaluation Criteria in Solid Tumors (RECIST) 1.1 [24]. Baseline assessment was performed within 2 weeks before the first-line chemotherapy. The chemotherapy response status was defined as complete response (CR), partial response (PR), stable disease (SD) and progressive disease (PD). Progression free survival (PFS) was calculated from the beginning of the first-line treatment until PD. Platelet counts, neutrophil, and lymphocyte values were collected before the initiation of first-line treatment.

### Statistical analysis

Student’s t test or Analysis of variance (ANOVA) were used to compare PLR, NLR and the clinical pathologic characteristics. Correlation between NLR and PLR, and the disease control rate (DCR; the rate of CR + PR + SD) between groups were assessed by Pearson’s correlation analysis. Association between differentiation score and other parameters were assessed by Chi-square test. Survival analysis was carried out using Kaplan-Meier methods and compared by the Log-rank test. The receiver operating characteristic (ROC) curve was applied to determine the optimal continuous variables cut-off points based on the largest Youden’s index. The prognostic prediction priority of different clinical parameters was compared by areas under the ROC curve (AUC). Univariate and multivariate Cox-regression analyses were performed to identify the independent predictors for PFS. All analyses were performed using the SPSS 23.0 statistical software program (IBM, USA). All statistical tests used in this study were two-sided and *P* < 0.05 was considered statistically significant.

## Results

### Clinicopathological characteristics

**Fig. 1:**
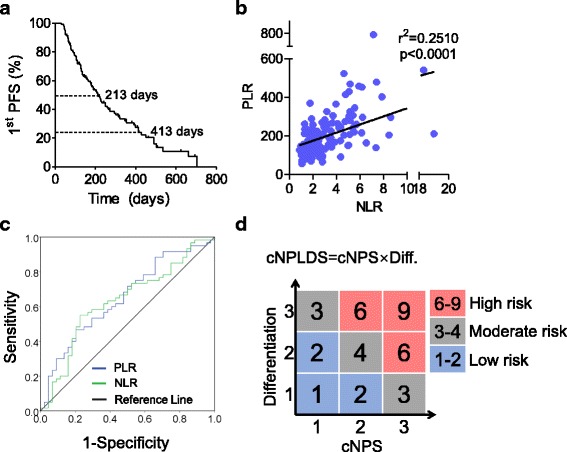
Parameter definitions. **a** Progression-free survival during the first-line chemotherapy (1st PFS) curve for all the 136 patients included in this study. **b** Correlation analysis between NLR and PLR. **c** ROC curve for NLR and PLR to determine the cut-off values at the median 1st PFS. **d** Schematic figures of cNPLDS and risk classifications

A total of 136 patients with advanced gastric cancer were included in this study, which satisfied the estimated sample size. Therein, 82 (60.3%) were male and 54 (39.7%) were female. The median age at diagnosis was 55 years (range 28–85 years). There were 90 (66.2%) patients were poorly differentiated, while 46 (33.8%) were moderate or well differentiated. The patients received regimens include Fluorouracil/Leucovorin/Oxaliplatin (FOLFOX), Capecitabine/Oxaliplatin (CapeOX), Paclitaxel or Docetaxel/Cisplatin (TP) and others. The PFS of all patients ranges from 30 to 703 days, and the median was 213 days (Fig. 1a). The median values of pre-treatment NLR and PLR were 2.60 and 181.14, respectively.

To investigate the associations of pre-treatment NLR and PLR with clinicopathologic characteristics of gastric cancer patients, comparisons between the different feature subgroups for NLR and PLR was carried out. We found that there was no significant difference of NLR and PLR in the different subgroups of gender, age, tumor differentiation and chemotherapy regimens (Table 1). The two systemic indices NLR and PLR have close positive correlation (r^2^ = 0.25, *P* < 0.0001; Fig. 1b).

**Table 1:** The clinical characteristics of 136 patients with advanced gastric cancer

Characteristic	Total *n* = 136 (%)	NLR		PLR	
		Mean ± SEM	*P value*	Mean ± SEM	*P value*
Gender			0.876		0.115
Male	82 (60.3%)	3.36 ± 0.30		191.66 ± 11.84	
Female	54 (39.7%)	3.43 ± 0.37		222.64 ± 16.00	
Age			0.446		0.966
≤55 years	74 (54.4%)	3.55 ± 0.37		204.34 ± 12.45	
>55 years	62 (45.6%)	3.20 ± 0.24		203.51 ± 15.06	
Differentiation			0.144		0.169
Poor	90 (66.2%)	3.59 ± 0.32		213.43 ± 10.70	
Moderate/Well	46 (33.8%)	2.99 ± 0.25		185.44 ± 19.07	
Chemotherapy regimen			0.158		0.964
FOLFOX	40 (29.4%)	3.77 ± 0.52		209.31 ± 16.29	
CapeOX	26 (19.1%)	3.94 ± 0.70		200.37 ± 26.76	
TP	21 (15.4%)	3.58 ± 0.42		210.15 ± 22.98	
Others	49 (36.1%)	2.71 ± 0.22		198.85 ± 15.84	

According to tumor differentiation, patients were stratified into well (score 1), medium (score 2) and poor (score 3) differentiated groups. Assessed by Chi-square assay, there were no significant association between differentiation score and other clinical characteristics, including gender, age, tumor differentiation and chemotherapy regimens (Table 2).

**Table 2:** The relationship between differentiation score and other clinical characteristics

Characteristic	Differentiation			
	Poor	Moderate	Well	*P value*
Gender				0.107
Male	49 (59.8%)	27 (32.9%)	6 (7.3%)	
Female	41 (75.9%)	12 (22.2%)	1 (1.9%)	
Age				0.284
≤55 years	53 (71.6%)	17 (23.0%)	4 (5.4%)	
>55 years	37 (59.7%)	22 (35.5%)	3 (4.8%)	
Chemotherapy regimen				0.310
FOLFOX	24 (60.0%)	14 (35.0%)	2 (5.0%)	
CapeOX	17 (65.4%)	9 (34.6%)	0 (0.0%)	
TP	16 (76.2%)	5 (23.8%)	0 (0.0%)	
Others	33 (67.3%)	11 (22.4%)	5 (10.2%)	

### The differentiation status and PLR are independent prognostic factors for the first-line chemotherapy response

To determine the cut-off points for the continuous variables, the ROC curves of NLR and PLR for the first-PFS were analyzed at the time point of overall median survial time (Day 213) (Fig. 1c). The optimal continuous variables cut-off points were determined by the largest Youden’s index. As results, the optimal cut-off points were 3.04 for NLR (sensitivity 55.0%, specificity 77.3%), and 223 for PLR (sensitivity 46.7%, specificity 79.5%). By this, the high value group was defined as greater than the cut-off points, whereas the low value was less than or equal to the cut-off points.

To find out the relationship between clinicopathological features and PFS, univariate analyses including gender, age, histological differentiation, NLR and PLR was performed. In results, histological differentiation, NLR and PLR were risk factors that significantly influenced PFS (Table 3). Next, histological differentiation, NLR and PLR were analyzed by multivariate Cox-regression model. We found that only differentiation status and PLR were the independent prognostic factors for PFS. Whereas, NLR had no independent prognostic value, though it was significantly associated with PFS in univariate analysis (Table 3). This made us curious whether combining these parameters together better predict the first-PFS.

**Table 3:** Univariate and multivariate analyses of clinical parameters for PFS prediction

Variables	Univariate analysis		Multivariate analysis	
	χ2	*P value*	Hazard ratio (95% CI)	*P value*
Gender (male vs. female)	0.041	0.838		
Age (≤55y vs. >55y)	0.317	0.574		
Differentiation (Poor vs. Moderate/Well)	11.79	< 0.001	1.775 (1.168–2.697)	0.007
NLR (≤3.04 vs. > 3.04)	18.03	< 0.001	1.027 (0.949–1.111)	0.506
PLR (≤223 vs. > 223)	10.66	0.001	1.003 (1.001–1.005)	0.010
cNPS (1 vs. 2 vs. 3)	19.50	< 0.001		
cNPLDS (1–2 vs. 3–4 vs. 6–9)	23.82	< 0.001		

### The cNPS and cNPLDS indicates poor prognosis of the first-line chemotherapy

According to the cut-off values, we used cNPS to reflect NLR and PLR status, which was defined as follows: patient with both elevated NLR (> 3.04) and PLR (> 223) was assigned a score of 3; patient with only either one elevated index was 2; patient with neither elevated was 1. Among these patients, 69 patients (50.7%) were of cNPS 1, 32 patients (23.5%) were of cNPS 2, and 35 patients (25.8%) were of cNPS 3. Since histological differentiation was independent prognostic factors for the first-PFS, we then additionally introduced a new parameter cNPLDS by multiplying cNPS and the differentiation score together (Fig. 1d). Therein, 27 patients (19.85%) were of cNPLDS 1–2, 55 (40.44%) were of 3–4, and 54 (39.71%) were of 6–9. In the univariate analyses, both high cNPS and high cNPLDS showed significantly correlation with shorter first-PFS (Table 2). Accordingly, we classified patient risk of progression by cNPLDS (Fig. 1d).

**Fig. 2:**
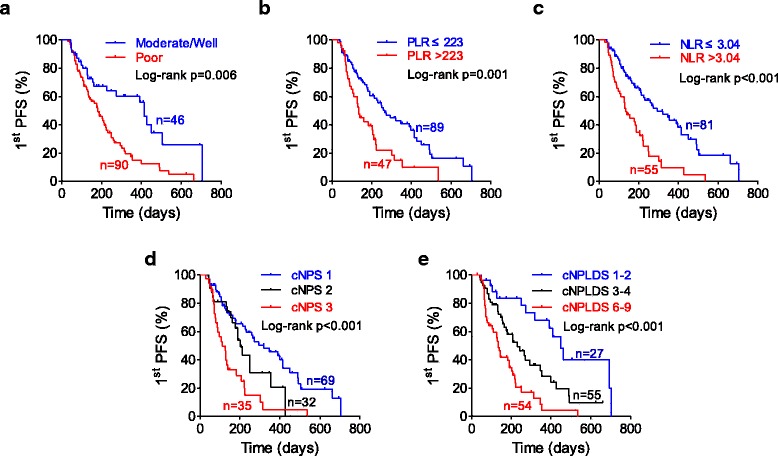
PFS analysis by Kaplan-Meier curves. Kaplan-Meier curves for 1st PFS in patients categorized by (**a**) histological differentiation, (**b**) NLR level, (**c**) PLR level (**d**) cNPS and (**e**) cNPLDS

All the significant parameters in univariate analysis were therefore evaluated in subsequent Kaplan-Meier survival analysis. Patients with poor differentiation gastric cancer had a significantly shorter PFS (median 182 days) than the moderate or well differentiations (median 413 days) (Fig. 2a). Patients with high NLR (> 3.04) or high PLR (> 223) were more easily to get chemoresistance during the first-line therapy with median PFS 134 vs. 293 days and 134 vs. 265 days, respectively (Figs. 2b and c). Patients of cNPS 3 had the poorest PFS (median 110 day), while those of cNPS 1 had most favorable PFS (median 320 day). The curve of cNPS 2 lay between them (median 202 day) (Fig. 2d). As to cNPLDS, patients of cNPLDS 1–2 (low risk) demonstrated the best prognosis (median 449 day), the cNPLDS 3–4 (moderate risk) ones had shorter first PFS (median 231 day), and cNPLDS 6–9 (high risk) ones were the worst (median 135 day) (Fig. 2e).

Table 4 list the first response assessment during the chemotherapy cycles. Most of the patients were SD 73.53%. Response outcome rates of CR and PR were 0% and 5.15% respectively in total. The DCR of higher NLR and PLR were significantly worse than the corresponding rest groups. For cNPS and cNPLDS, DCR decreased progressively along with the score elevation. However, no statistical significance was reached when grouped by tumor differentiation status.

**Table 4:** Chemotherapy response assessment

	Differentiation status		PLR		NLR		cNPS			cNPLDS		
	well/moderate	poor	≤223	> 223	≤3.04	> 3.04	1	2	3	1–2	3–4	6–9
Total	46	90	89	47	81	55	69	32	35	27	55	54
CR	0 (0%)	0 (0%)	0 (0%)	0 (0%)	0 (0%)	0 (0%)	0 (0%)	0 (0%)	0 (0%)	0 (0%)	0 (0%)	0 (0%)
PR	3 (6.52%)	4 (4.44%)	4 (4.49%)	3 (6.38%)	3 (3.70%)	4 (7.27%)	2 (2.90%)	3 (9.38%)	2 (5.71%)	2 (7.41%)	1 (1.82%)	4 (7.41%)
SD	36 (78.26%)	64 (71.11%)	72 (80.89%)	28 (59.57%)	68 (83.95%)	32 (58.18%)	59 (85.51%)	22 (68.75%)	17 (48.57%)	23 (85.19%)	46 (83.64%)	31 (57.41%)
PD	7 (15.21%)	22 (24.44%)	13 (14.61%)	16 (34.04%)	10 (12.34%)	19 (34.55%)	8 (11.59%)	7 (21.87%)	14 (40%)	2 (7.41%)	8 (14.55%)	19 (35.19%)
DCR	84.78%	75.56%	85.39%	65.96%	87.65%	65.45%	88.40%	78.13%	60%	92.59%	85.45%	64.81%
P	0.214		0.008		0.002		0.004			0.004		

### The cNPLDS is superior in predicting the first-line chemotherapy response

**Fig. 3:**
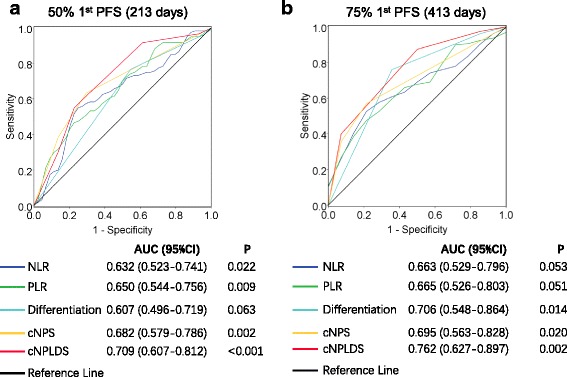
Comparing prognostic prediction priority using ROC curves. ROC curves for histological differentiation, NLR, PLR, cNPS and cNPLDS at (**a**) the median and (**b**) the third-quarter of 1st PFS

To assess prognostic accuracy, we compared the AUC at the median (Day 213) of the first-PFS in all patients. The AUC for differential status, NLR and PLR were 0.607, 0.632 and 0.650, respectively. When integrating NLR and PLR, the classification was greatly improved by cNPS with an AUC of 0.682. Additionally, when taking differential status into account together with cNPS, cNPLDS further improved AUC to 0.709 (Fig. 3a). Moreover, we also compare the AUC at the third quarter (Day 413) of PFS to see the longer effect. Similarly, cNPLDS (AUC = 0.762) is superior to differential status (AUC = 0.706), NLR (AUC = 0.663) and PLR (AUC = 0.665) alone, and integrated cNPS (AUC = 0.695) (Fig. 3b). The cNPLDS had the higher AUC value for the third quarter PFS than the median, suggesting cNPLDS was more accurate in the long-term prognostic prediction.

## Discussion

Chemotherapy is the main treatment for advanced unresectable gastric cancer, and the sensitivity to first-line chemotherapy has a direct impact on overall survival prognosis [25]. In this study, we introduce a novel prognostic parameter cNPLDS, integrating systemic NLR and PLR and local tumor differentiation status to predict the PFS of the first-line chemotherapy in advanced gastric cancer. We proved that the AUC of cNPLDS is larger than NLR, PLR, and cNPS, showing its priority in predicting prognosis. In addition, as an adverse prognostic factor, cNPLDS covers more high-risk population, and further refines the hazard classification. Moreover, cNPLDS is an economical clinical tool, which just depends on the basic diagnostic indices and dispenses with additional examinations. Hence, it is easy to apply in the less developed areas, where gastric cancer is generally of high prevalence and high mortality.

Previously, many studies used peripheral blood routine examination indices, e.g. NLR or PLR, and their derived cNPS, as predictors for curative resected gastric cancer recurrence or overall survival [17–19, 23]. However, studies about these parameters for chemotherapeutic response prediction in advanced unresectable gastric cancer are not too much [26–28]. Recently, a study based on the REAL-2 trail advanced oesophago-gastric cancer population reported that elevated NLR status demonstrate a significant negative prognostic effect on the first-line chemotherapy response [26]. It was also demonstrated that F-NLR score, a combining score of NLR and fibrinogen, predicted the therapeutic response of advanced gastric cancer to chemotherapy or chemoradiotherapy [28]. Interestingly, another study of advanced gastric cancer showed an association between the mismatch repair system status and NLR, which both influence the PFS and overall survival (OS) [27]. This suggested that the great histopathological heterogeneity should not be overlooked. Therein, tumor differentiation is also one of the most basic histopathological index. Given these, the novel cNPLDS was introduced, which reflects both systemic immunological status and local pathological characteristics. In this study, such combination was showed to improve the prediction efficiency. Moreover, since cNPLDS is a complicated prognostic parameter, we believe it would be more stable than single index. No statistical correlation was found between tumor differentiation and NLR or PLR, which suggested they were two relatively independent sets of mechanism that both influence therapeutic response. This is probably another reason why cNPLDS is superior to differential status, NLR, PLR or integrated cNPS.

It is known that inflammation accompanies along with cancer progression [10]. In addition to local inflammatory response, systemic inflammatory response is also an important prognostic factor in cancer. Many experimental studies documented that neutrophils, platelets and lymphocytes participate in the cancer generation or malignancy progression [29–31]. It was reported that local cancer associated neutrophils of N2 phenotypes promote tumor growth and invasion by altering the extracellular matrix and inhibiting the function of other immune cells [31]. Activated platelets, which release various growth factors and cytokines, promote tumor metastasis [32, 33], angiogenesis [32, 34], and chemoresistance [32, 35]. Platelets can help the circulating tumor cells forming cancer embolus, and protect them from the immunocytes [32, 36]. Recently studies showed that platelet activation triggered platelet-neutrophil interaction and alters the immunocyte subpopulations [37]. In this study, we found a close correlation between NLR and PLR, suggesting that peripheral circulating neutrophils and platelets may play a synergetic effect on primary chemoresistance. Conversely, lymphocytes possess anti-tumor effect [38]. We reported that platelets could enhance the differentiation and cytokine production of CD4^+^ T lymphocytes [39], which could be regarded as a physiological balance. However, when the balance is broken, like the increase of the neutrophils or platelets but decrease of lymphocytes, the systemic immunity will turn abnormal. This probably explains why high NLR, PLR or cNPS patients has shorter PFS.

In the cNPLDS, tumor differentiation status is a relative steady composition, while other elements of neutrophils, platelets and lymphocytes were dynamic. This made us curious whether intervention of the latter might cover the deficiency of poor differentiation status on prognosis. Recently, increasing clinical data showed that tartigeting-PD-1 or PD-L1 treatment plus chemotherapy received more favorable efficiency [40], suggesting activating lymphocyte may be a promising approach for chemotherapy optimizing. Besides lymphocytes activation, we also wonder whether inhibiting neutrophils or platelets designedly to certain level or suppressing their activities may improve the therapeutic effect. Few studies explored pharmacological inhibition on neutrophils, which is probably because of catastrophic side effects, such as agranulocytosis or consequent sever infection. However, recent in vitro and in vivo study showed that some antiplatelet activation drugs, such as aspirin [41], P2Y12 inhibitor [42], and Integrin β3 inhibitors [43], may play tumor suppressive roles. Although some clinical trials reported the value of aspirin in cancer prevention and improving forward prognosis [44–46], there were also some negative results documented [47, 48]. On the other hand, there were no good indicators to determine which subgroups of patients might benefit from platelet intervention. This probably resulted in the trail failure. In this study, cNPLDS is introduced as a prognostic factor integrating platelets, neutrophils, lymphocytes and tumor differentiation, and we suppose that those high cNPLDS patients may probably benefit from the pharmacological interventions. However, this need further studies and larger data to support.

## Conclusion

In conclusion, our study demonstrates the novel parameter cNPLDS is superior to NLR or PLR alone, or their derived cNPS. The cNPLDS is a continent and economical prognostic predictor for the first-line chemotherapeutic response in advanced unresectable gastric cancer.

## Abbreviations

ANOVA: Analysis of variance
AUC: Areas under the ROC curve
cNPLDS: Combined Neutrophil/platelet/lymphocyte/differentiation Score
cNPS: Combined NLR-PLR score
CR: Complete response
CSCO: The Chinese Society of Clinical Oncology
ESMO: The European Society of Medical Oncology
NCCN: The National Comprehensive Cancer Network (US)
NLR: Neutrophil-to-lymphocyte ratio
PD: Progressive disease
PFS: Progression free survival
PLR: Platelet-to-lymphocyte ratio
PR: Partial response
RECIST: The Response Evaluation Criteria in Solid Tumors
ROC curve: Receiver operating characteristic curve
SD: Stable disease
